# Microbial‐derived Exerkines preserve muscle size and function during disuse in aged mice in a sex‐dependent manner

**DOI:** 10.14814/phy2.71137

**Published:** 2026-10-05

**Authors:** Jainil Daredia, Taylor R. Valentino, Ahmed Ismaeel, Yuan Wen, John J. McCarthy, Benjamin I. Burke

**Affiliations:** ^1^ Department of Physiology, College of Medicine University of Kentucky Lexington Kentucky USA; ^2^ Center for Muscle Biology, College of Health Sciences University of Kentucky Lexington Kentucky USA; ^3^ Buck Institute for Research on Aging Novato California USA; ^4^ Department of Anatomy, Physiology and Pharmacology, College of Veterinary Medicine Auburn University Auburn Alabama USA; ^5^ Division of Biomedical Informatics, Department of Internal Medicine, College of Medicine University of Kentucky Lexington Kentucky USA

**Keywords:** aging, disuse atrophy, muscle function, pipecolic acid, succinate

## Abstract

Disuse‐induced muscle atrophy is a critical clinical challenge in older adults. Existing countermeasures have largely failed to preserve muscle size and function, underscoring the need for novel therapeutic strategies. We recently identified pipecolic acid and succinate (PAS) as microbial‐derived exerkines that prevent disuse‐induced atrophy and preserve muscle function in adult mice. Here, we tested whether PAS similarly protects aged muscle. Aged male and female mice received vehicle (VEH) or PAS via drinking water during 10 days of unilateral hindlimb casting. PAS did not preserve soleus or plantaris mass, but casted gastrocnemius mass was greater in PAS‐ than VEH‐treated mice irrespective of sex. PAS attenuated the loss of soleus and plantaris fiber cross‐sectional area, with the largest effect in the female soleus. PAS enhanced soleus contractile function in a sex‐specific manner: females exhibited greater absolute force and rate of force development, and males greater fatigue resistance. Gastrocnemius citrate synthase activity was greater in PAS‐ than VEH‐treated females but not males, mirroring the female‐specific preservation of soleus fiber size and function. These sexually dimorphic responses highlight microbial‐derived metabolites as a novel therapeutic avenue for preserving muscle function during disuse in aging and underscore the importance of considering sex as a biological variable.

## 1 INTRODUCTION

Skeletal muscle is highly sensitive to disuse; even brief periods of physical inactivity, hospitalization, or injury‐related immobilization induce notable muscle atrophy and functional decline. Within just 2 weeks of unloading in young adults, muscle strength decreases by ~23%, accompanied by losses of ~0.6% of muscle cross‐sectional area (CSA) per day (Wall et al., 2013). Such episodes of disuse are disproportionately more common in older adults, among whom the atrophic consequences are far more severe (Alley et al., 2010; Cruz‐Jentoft et al., 2019; Deschenes et al., 2008). Critically, declines in muscle strength and power most directly threaten functional independence and longevity in the aging population (Covinsky et al., 2003). Even as few as 4 days of disuse are sufficient to cause substantial declines in lower extremity strength and functional capacity in older adults (Elam et al., 2022; Hvid et al., 2014; Kortebein et al., 2008; Rommersbach et al., 2020). Kortebein and colleagues demonstrated that 10 days of bed rest resulted in a 13.2% decline in knee extensor strength, a 14% reduction in stair climbing power, and a 12% decrease in aerobic capacity in healthy older adults (Kortebein et al., 2007; Kortebein et al., 2008). Similarly, 2 weeks of disease‐related immobilization or unilateral whole‐leg casting has been shown to result in a significant loss of thigh muscle mass, reductions in isometric knee extension strength, and impaired postural control in older patients (Elam et al., 2022; Rommersbach et al., 2020). These detriments are not isolated events but components of a vicious cycle, wherein aging increases fall risk, falls lead to fractures and further immobilization, and immobilization drives accelerated muscle loss and frailty, leaving older adults particularly vulnerable to falls, functional decline, and loss of independence (Mallery & Shetty, 2026).

A further challenge in preserving muscle mass and/or function during disuse is the limited practicality of exercise interventions in elderly populations. In particular, the fear of injury and falling, preexisting health conditions, lack of motivation, and financial costs act as potent exercise deterrents (Zhong et al., 2024). Even when resistance exercise can be performed, an age‐related reduction in sensitivity to exercise and nutritional stimuli, termed anabolic resistance, substantially limits the efficacy of such interventions in preserving skeletal muscle size and function in older adults (Brook et al., 2016; Drummond et al., 2008; Marshall et al., 2020; McKendry et al., 2024). Disuse itself can further exacerbate anabolic resistance, as even an acute immobilization period has been shown to blunt the protein synthesis machinery required for recovery after remobilization (Wall et al., 2016). Together, these findings highlight the urgent need for effective interventions capable of both preserving muscle size and function during immobilization in older adults.

Several countermeasures to disuse‐induced muscle loss during immobilization have been investigated, including neuromuscular electrical stimulation, protein and amino acid supplementation, β‐hydroxy‐β‐methylbutyrate, and omega‐3 fatty acids, yet interventions that preserve both muscle strength and physical function in older adults remain limited (Deutz et al., 2013; Dirks, Wall, Snijders, et al., 2014; McGlory et al., 2019; Reidy et al., 2017; Suetta et al., 2008). Our recent work identified pipecolic acid and succinate (PAS) as key microbial‐derived exerkines whose oral co‐administration proved effective at preventing disuse‐induced skeletal muscle atrophy and preserving muscle function in adult mice (Burke et al., 2026). Given the ability of PAS to preserve skeletal muscle size and function during disuse and the heightened susceptibility of aged muscle to disuse‐induced atrophy, we hypothesized that oral PAS administration would similarly preserve skeletal muscle size and function in aged mice subjected to disuse. To test this hypothesis, 22‐ to 25‐month‐old male and female C57BL/6JN mice underwent 10 days of unilateral hindlimb casting and received either vehicle (VEH) or PAS via drinking water throughout the immobilization period. Following casting, hindlimb muscles were evaluated for muscle mass, histological fiber morphology, ex vivo contractile function, and mitochondrial content.

## 2 MATERIALS AND METHODS

### 2.1 Animals

22‐ to 25‐month‐old male and female C57BL/6JN mice from the NIA aged rodent colony were used for all experiments. Muscle mass, histology, and citrate synthase activity were assessed in one cohort (male, *n* = 6/treatment; female, *n* = 5/treatment); ex vivo contractile function was assessed in a second cohort (*n* = 5/sex/treatment). Mice were singly housed in a temperature and humidity‐controlled room on a 14 h:10 h light–dark cycle with ad libitum access to food (2918 Teklad Global; Inotiv, West Lafayette, IN) and water. Mice were unilaterally immobilized via casting for 10 days as previously described (Burke et al., 2026). Briefly, one limb was enclosed in a 3D‐printed plastic cast in gentle flexion at the knee and dorsiflexion at the ankle, while the contralateral limb remained free. Mice were randomly assigned to receive VEH (2% sucrose) or PAS (189.7 ± 79.5 mg/kg/d pipecolic acid [50‐194‐7204; Fisher Scientific, Waltham, MA] and 94.8 ± 39.7 mg/kg/d succinate [S7501; Sigma‐Aldrich, Burlington, MA] in 2% sucrose) via drinking water throughout the immobilization period (Figure 1a). Body mass was recorded at the start and end of the immobilization period, and water intake was recorded daily. Sucrose was included in both solutions to improve palatability; daily water intake was nonetheless 11.5% lower in PAS‐treated than VEH‐treated mice. Mice were euthanized after the immobilization period by cervical dislocation under deep anesthesia (2% isoflurane). All animal procedures were approved by the Institutional Animal Care and Use Committee at the University of Kentucky.

**FIGURE 1:**
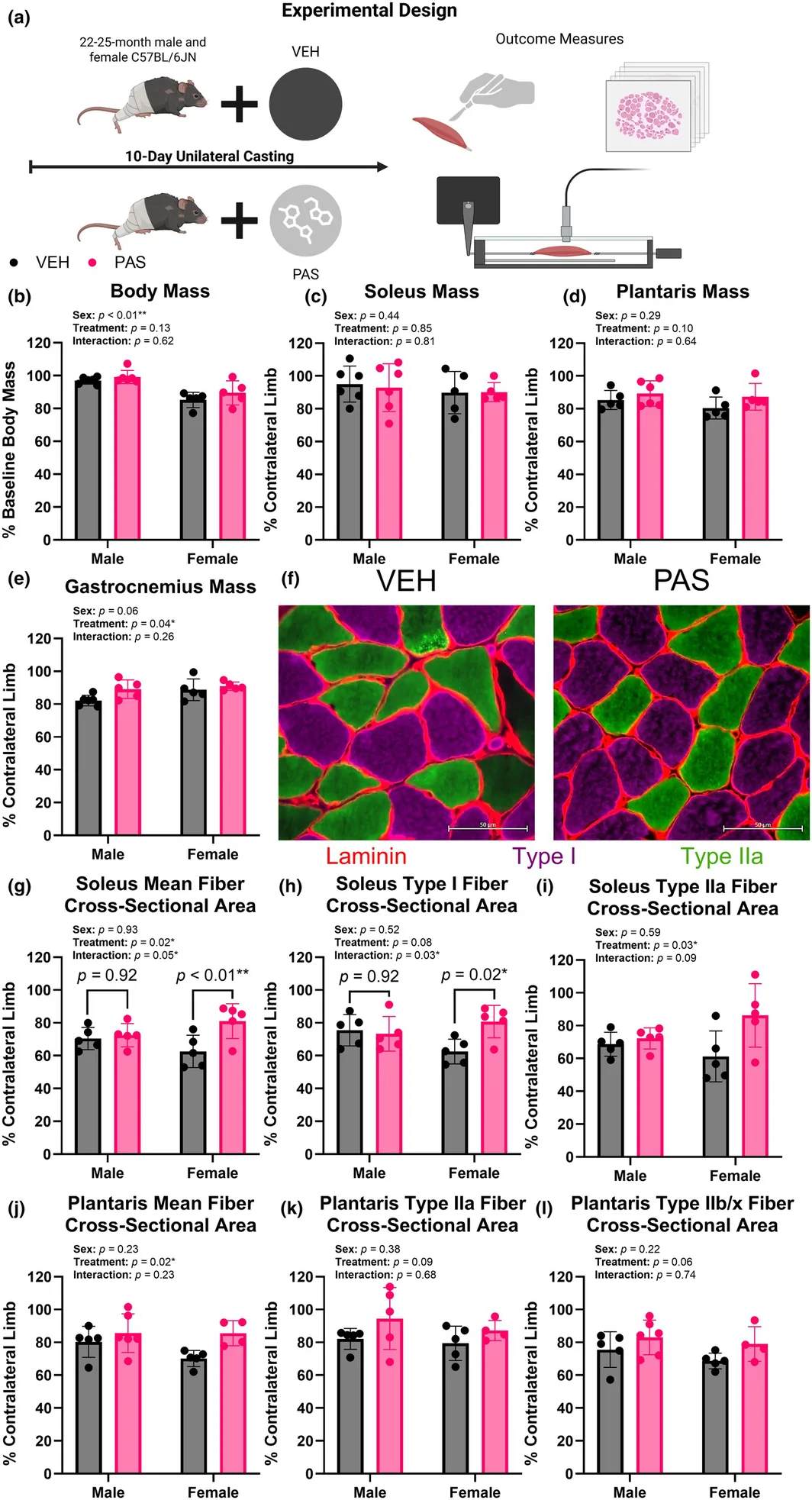
PAS attenuates disuse‐induced loss of muscle size in aged mice. (a) Experimental design; 22‐ to 25‐month‐old male and female C57BL/6JN mice underwent 10 days of unilateral hindlimb casting while receiving vehicle (VEH) or pipecolic acid and succinate (PAS) via drinking water. (b) Body mass at the end of immobilization expressed as a percentage of baseline body mass (*n* = 5–6/group). (c) Soleus, (d) plantaris, and (e) gastrocnemius mass (*n* = 5‐6/group) of the casted limb expressed as a percentage of the contralateral control limb. (f) Representative immunofluorescence images of casted soleus cross‐sections from VEH‐ and PAS‐treated female mice stained for laminin (red) and myosin heavy chain Type I (purple) and Type IIa (green); scale bar = 50 μm. (g) Mean, (h) Type I, and (i) Type IIa fiber cross‐sectional area of the casted soleus (*n* = 5/group), expressed as a percentage of the contralateral control limb. (j) Mean, (k) Type IIa, and (l) Type IIb/x fiber cross‐sectional area of the casted plantaris (*n* = 4–6/group), expressed as a percentage of the contralateral control limb. All data were analyzed by two‐way ANOVA (sex × treatment); main effect and interaction *p* values are displayed on each panel. Treatments were compared within each sex where the sex × treatment interaction was significant, and the resulting *p* values are shown above the relevant comparison. Data are presented as mean ± SD. *p < 0.05; **p < 0.01.

### 2.2 Muscle phenotyping

Upon euthanasia, the soleus, plantaris, and gastrocnemius muscles from both the casted and contralateral limb were carefully excised and weighed; one plantaris (VEH) and one gastrocnemius (PAS) were removed from the male group due to dissection variability. To assess muscle phenotype, the soleus and plantaris from the casted and contralateral limb were embedded in Tissue‐Tek Optimal Cutting Temperature compound (4583; Sakura Finetek, Torrance, CA, USA) and frozen in liquid nitrogen‐chilled isopentane. Samples were sectioned into 8‐μm‐thick cross‐sections at the muscle mid‐belly and dried at room temperature. Sections from *n* = 88 muscles (male and female, VEH and PAS, soleus and plantaris, casted and contralateral) were prepared across eight slides organized by muscle and sex, with each slide containing all four treatment‐casting combinations. Two male solei (VEH, PAS), one male plantaris (VEH), and one female plantaris (PAS) were removed due to poor section quality. Sections were incubated with primary antibodies against laminin (1:200; L9393, Sigma‐Aldrich), myosin heavy chain Type I (1:200; BA‐D5, Developmental Studies Hybridoma Bank, Iowa City, IA), and myosin heavy chain Type IIa (1:200; SC‐71, Developmental Studies Hybridoma Bank) prepared in 1% BSA with 0.1% Triton X‐100 for 90 min at room temperature. Sections were then incubated with fluorescence‐conjugated secondary antibodies (1:250; A‐21442, A‐21242, A‐21121; ThermoFisher Scientific, Waltham, MA) in 0.1% Triton X‐100 for 75 min. Unstained myofibers were categorized as Type IIb/x fibers. Images of entire sections were acquired using an Olympus BX61VS upright fluorescence microscope (Evident Scientific, Bethlehem, PA) at 20× magnification and analyzed using MyoVision software (Viggars et al., 2022; Wen et al., 2018) for automated analysis of fiber‐type‐specific CSA. Muscle mass and fiber CSA from the casted limb were expressed as a percentage of the contralateral control limb.

### 2.3 Ex vivo muscle function

Immediately following excision, ex vivo muscle function of the soleus muscle was assessed using established protocols (Burke et al., 2026; Minchew et al., 2022). To minimize the interval between excision and evaluation and to avoid time‐, temperature‐, and oxygen‐dependent changes in contractile output (Barclay, 2005; Edwards et al., 2007; Segal & Faulkner, 1985; Zhang et al., 2006), only the casted soleus was analyzed. Briefly, the soleus was mounted in an organ bath (820MO; Danish Myo Technology, Essen, Germany) containing room temperature oxygenated (95% O_2_/5% CO_2_) Krebs‐Ringer buffer (in mM: 119 NaCl, 5.0 KCl, 5.0 NaHCO_3_, 1.25 CaCl_2_, 1.0 KH_2_PO_4_, 10 HEPES, 1.0 MgSO_4_; pH 7.2). After establishing optimal length, the muscle was stimulated at three‐minute intervals at 10, 20, 40, 60, 80, 100, and 120 Hz (biphasic, 20 V, 0.2 ms pulse width, 300 ms trains; CS4+ Stimulator; Danish Myo Technology). Fatigability was then assessed by stimulating the muscle 150 times at 60 Hz every 2 s. Force output was recorded using LabChart software (v.8.1.19) and analyzed using MATLAB (v.R2024b Update 5). One male soleus (VEH) was excluded from all ex vivo analyses, and two female solei (PAS) were excluded from the fatigue protocol only, owing to muscle or tendon damage that obscured accurate force readings. Absolute force output (mN) and rate of force development (RFD; N/s) were reported.

### 2.4 Citrate synthase activity

Citrate synthase activity was measured in gastrocnemius homogenates by enzymatic assay as previously described (Burke et al., 2026; Ismaeel et al., 2023). Equal protein amounts (20 μg) were suspended in a buffer containing 100 mM Tris (pH 8.0), 0.1 mM 5,5′‐dithiobis (2‐nitrobenzoic acid) (D8130; Sigma‐Aldrich), 0.3 mM acetyl‐CoA (A2056; Sigma‐Aldrich), and 0.5 mM oxaloacetic acid (O4126; Sigma‐Aldrich). The change in absorbance at 412 nm was recorded over 10 min using a Cytation 5 multimode reader (BioTek, Winooski, VT). Activity was calculated as U/mg protein = (Δabsorbance/Δtime in minutes) / (13.6 × 0.625 × mg protein).

### 2.5 Statistics

Two‐way ANOVAs (sex and treatment) were used to test differences in body mass, muscle mass, fiber CSA, and citrate synthase activity. Three‐way ANOVAs were used to test differences in force production and RFD (frequency, sex, and treatment) and fatigue resistance (contraction number, sex, and treatment). Where the sex × treatment interaction was significant, treatments were compared within each sex (Šídák's multiple comparisons test for the two‐way analyses; within‐sex ANOVA for the three‐way analyses); where it was not, main effects were interpreted and no pairwise comparisons were performed. Significance was set at α = 0.05. All statistical analyses were performed using GraphPad Prism v.11.0.1 for Windows (GraphPad Software, La Jolla, CA).

## 3 RESULTS

### 3.1 Body and muscle mass

Casting was well tolerated, although body mass declined over the immobilization period, particularly in females. There was a main effect of sex on the change in body mass during casting (*p* < 0.01), such that females lost more body mass than males (87.4 ± 6.2% vs. 98.1 ± 3.3% relative to baseline), with no effect of treatment (*p* = 0.13) or interaction (*p* = 0.62; Figure 1b). To determine whether PAS preserved muscle mass during immobilization, soleus, plantaris, and gastrocnemius mass of the casted limb was expressed as a percentage of the contralateral control limb. Soleus mass of the casted limb did not differ by sex (*p* = 0.44) or treatment (*p* = 0.85), and there was no sex × treatment interaction (*p* = 0.81; Figure 1c). Plantaris mass was likewise unaffected by sex (*p* = 0.29) or treatment (*p* = 0.10), with no interaction (*p* = 0.64; Figure 1d). In the gastrocnemius, however, there was a main effect of treatment (*p* = 0.04), such that PAS‐treated mice retained a greater proportion of gastrocnemius mass in the casted limb than VEH‐treated mice (90.0 ± 4.2% vs. 85.2 ± 5.9% of the contralateral control limb) irrespective of sex (interaction *p* = 0.26), although the effect of sex approached significance (*p* = 0.06), with males tending to lose more gastrocnemius mass than females (Figure 1e). Collectively, these findings indicate that PAS attenuated the casting‐induced reduction in gastrocnemius mass of both sexes while leaving soleus and plantaris mass unchanged.

### 3.2 Fiber cross‐sectional area

To characterize the morphological effects of PAS during disuse at the fiber level, mean and fiber‐type‐specific CSA were quantified in the soleus and plantaris and expressed as the casted limb relative to the contralateral control limb. In the soleus, there was a sex × treatment interaction for mean fiber CSA (*p* = 0.05), such that PAS‐treated females retained a larger mean fiber CSA than VEH‐treated females (*p* < 0.01), whereas mean fiber CSA did not differ between treatments in males (*p* = 0.92; Figure 1f,g). The same pattern was evident for Type I fibers, where a sex × treatment interaction (*p* = 0.03) reflected a greater Type I fiber CSA in PAS‐ than VEH‐treated females (*p* = 0.02) with no treatment difference in males (*p* = 0.92; Figure 1h). For Type IIa fibers, there was a main effect of treatment (*p* = 0.03), such that PAS‐treated mice retained a greater Type IIa fiber CSA than VEH‐treated mice, with no effect of sex (*p* = 0.59; Figure 1i); a trending sex × treatment interaction (*p* = 0.09) suggests this effect was driven largely by females.

To determine whether the fiber‐level effects of PAS extended to a muscle of contrasting fiber‐type composition and metabolic profile, the same analyses were performed in the plantaris. Mean plantaris fiber CSA showed a main effect of treatment (*p* = 0.02), with PAS‐treated mice retaining a greater mean fiber CSA in the casted limb than VEH‐treated mice independent of sex (sex effect *p* = 0.23; interaction *p* = 0.23; Figure 1j). Similar trending effects were observed for Type IIa (treatment, *p* = 0.09) and Type IIb/x fibers (treatment, *p* = 0.06), and neither fiber type showed an effect of sex (*p* = 0.38 and *p* = 0.22, respectively) or an interaction (*p* = 0.68 and *p* = 0.74, respectively; Figure 1k,l). Collectively, these findings demonstrate that PAS attenuated the casting‐induced reduction in fiber CSA in both the soleus and plantaris of aged mice, with sex‐specific effects on the soleus.

### 3.3 Ex vivo muscle function

To determine whether PAS preserved contractile function in the casted soleus of aged mice, force production, RFD, and fatigue resistance were assessed in the immobilized limb. The effect of PAS on force production depended on sex (sex × treatment interaction, *p* < 0.01; Figure 2a). PAS‐treated females produced greater absolute force than VEH‐treated females across the frequency range (*p* < 0.01), whereas force production did not differ between PAS‐ and VEH‐treated males (*p* = 0.34). RFD followed the same pattern (sex × treatment interaction, *p* < 0.01), with PAS‐treated females developing force more rapidly than VEH‐treated females (*p* < 0.01) and no treatment difference in males (*p* = 0.25; Figure 2b). Fatigue resistance showed the converse pattern (sex × treatment interaction, *p* < 0.01): PAS‐treated males sustained a greater proportion of their initial force than their VEH‐treated counterparts (*p* < 0.01), whereas females did not differ (*p* = 0.94; Figure 2c). Collectively, these findings demonstrate that PAS enhanced ex vivo contractile function of the casted soleus in aged mice, with greater force production and RFD in females and greater fatigue resistance in males.

**FIGURE 2:**
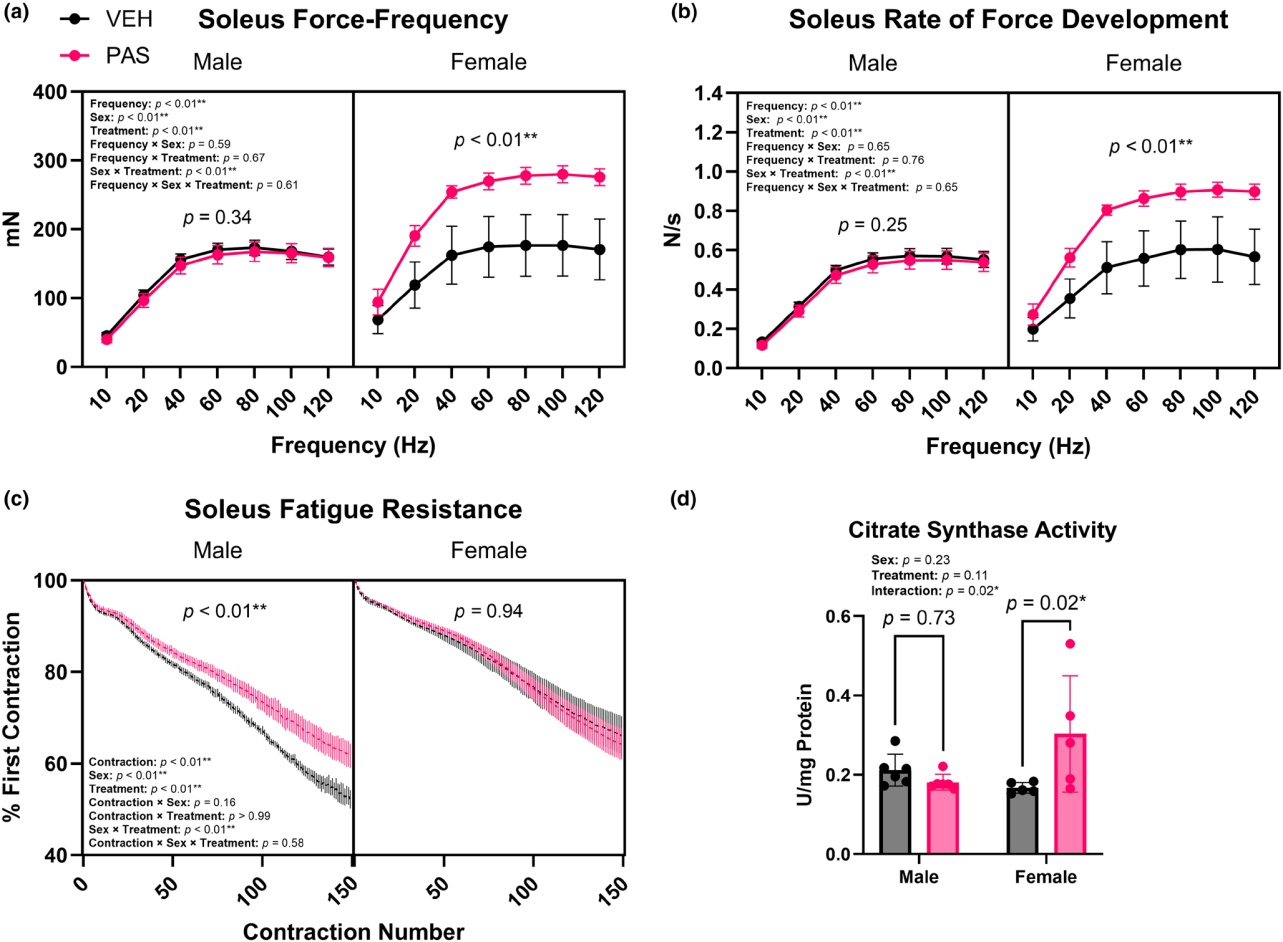
PAS treatment during disuse produces sex‐ and outcome‐specific improvements in soleus contractile function and mitochondrial content in aged mice. (a) Absolute force and (b) rate of force development across stimulation frequencies (10–120 Hz), and (c) fatigue resistance across 150 repeated contractions, in the casted soleus of male and female mice (*n* = 3–5/group) treated with vehicle (VEH) or pipecolic acid and succinate (PAS). (d) Citrate synthase activity in the casted gastrocnemius as an index of mitochondrial content (*n* = 5–6/group). Force, rate of force development, and fatigue resistance were analyzed by three‐way ANOVA (sex × treatment × frequency or contraction number); citrate synthase activity was analyzed by two‐way ANOVA (sex × treatment). Main effect and interaction *p* values are displayed on each panel. Treatments were compared within each sex where the sex × treatment interaction was significant, and the resulting *p* values are shown above the relevant comparison. Data are presented as mean ± SEM in a–c; mean ± SD in d. *p < 0.05; **p < 0.01.

### 3.4 Citrate synthase activity

To determine whether PAS altered mitochondrial content, citrate synthase activity was measured in the casted gastrocnemius. There was a sex × treatment interaction (*p* = 0.02), such that PAS‐treated females exhibited greater citrate synthase activity than VEH‐treated females (*p* = 0.02), whereas activity did not differ between treatments in males (*p* = 0.73; Figure 2d). These findings indicate that the effect of PAS on mitochondrial content in aged muscle was female specific, paralleling the sex specificity observed for soleus fiber size and contractile function.

## 4 DISCUSSION

Disuse atrophy in aged muscle represents a clinically significant challenge, as older individuals are disproportionately vulnerable to accelerated muscle loss and functional decline during periods of immobilization and demonstrate impaired recovery compared to younger counterparts (Alley et al., 2010; Deschenes et al., 2008; Hvid et al., 2010; Hvid et al., 2014). In the present study, we examined whether oral supplementation with PAS could preserve skeletal muscle size and function in aged mice subjected to 10 days of unilateral hindlimb casting. PAS attenuated the loss of soleus and plantaris fiber size and enhanced soleus contractile function in sex‐specific and outcome‐dependent manners. These findings demonstrate that the protective effects of PAS on muscle size and contractile function seen in adult mice extend to aged mice, though in a sexually dimorphic pattern. Functional preservation of this kind has not been achieved by existing interventions targeting disuse atrophy in aging.

### 4.1 PAS partially attenuates muscle mass loss with disuse in aged mice

Treatment with PAS during 10 days of unilateral hindlimb casting did not significantly attenuate casting‐induced reductions in soleus or plantaris muscle mass in aged mice, consistent with our previous findings in adult mice, in which PAS preserved fiber size without significantly altering whole‐muscle mass (Burke et al., 2026). However, PAS attenuated the loss in gastrocnemius mass, the largest of the three muscles and the one sustaining the greatest absolute mass loss. It is possible that a protective effect of PAS is most readily detected where the atrophic stimulus produces the largest absolute deficit, though we cannot exclude that it reflects muscle‐specific differences in metabolite uptake or fiber‐type composition. Nevertheless, the failure of PAS to protect against mass loss in the soleus and plantaris is consistent with the broader literature demonstrating the difficulty of preserving muscle mass during disuse in older populations. Protein and amino acid supplementation strategies, among the most studied countermeasures, have largely failed to prevent muscle mass loss during bed rest or limb immobilization in older adults (Churchward‐Venne et al., 2026; Dirks, Wall, Nilwik, et al., 2014). This pattern of failure likely reflects anabolic resistance in aged muscle, whereby the response to both nutritional and mechanical stimuli is substantially blunted, posing a significant challenge to both muscle mass preservation and recovery in this population (Brook et al., 2016; Wall et al., 2015).

### 4.2 PAS attenuates disuse‐induced fiber atrophy in both the soleus and plantaris

In contrast to the findings for muscle mass, PAS treatment protected fiber CSA in both the soleus and plantaris. The discrepancy between whole muscle mass and fiber CSA outcomes is not unexpected, as whole‐muscle mass includes contributions from non‐contractile tissue, extracellular fluid, and connective tissue that may obscure fiber‐level changes. In the soleus, analyses revealed a sex‐specific response at the muscle fiber level such that PAS appeared to be more protective against disuse‐induced reductions in fiber CSA in females compared to males. In light of prior reports suggesting that females exhibit greater susceptibility to disuse‐induced muscle atrophy than males, the female specificity of this morphological response is particularly interesting. Evidence from both rodent and human studies suggests that females experience greater relative muscle mass and fiber CSA losses during immobilization compared to their male counterparts (Rosa‐Caldwell et al., 2021; Rosa‐Caldwell & Greene, 2019; Tsitkanou et al., 2023; Wu et al., 2022). The absence of a comparable fiber‐level protective effect in PAS‐treated males suggests that PAS may differentially influence disuse‐related morphological adaptations in a sex‐dependent manner. The mechanisms underlying this divergence warrant dedicated mechanistic investigation in future studies.

The inclusion of the plantaris allowed the effects of PAS to be evaluated in muscles of contrasting fiber‐type composition and metabolic profile. Given that succinate promotes an oxidative, slow‐fiber program in skeletal muscle via SUCNR1 signaling (Wang et al., 2020), a benefit restricted to the predominantly slow, oxidative soleus might have been anticipated. Instead, PAS attenuated fiber atrophy in the predominantly fast, glycolytic plantaris as well, with similar effects for both Type IIa and Type IIb/x fibers. This suggests that the fiber‐level protection conferred by PAS during disuse is not confined to oxidative fibers and is therefore unlikely to be explained solely by succinate‐driven oxidative remodeling. Pipecolic acid, whose actions in skeletal muscle remain largely uncharacterized, may contribute to the fiber‐type‐independent component of this response, and dissecting the individual contributions of the two metabolites is an important next step.

### 4.3 Sex‐specific preservation of muscle function

In the present work, PAS‐treated females produced significantly higher absolute force and RFD across stimulation frequencies compared to VEH‐treated females, while there were no differences in males. This is particularly striking given that females not only appear more susceptible to disuse atrophy but also experience greater losses in muscle strength during immobilization relative to males (Yasuda et al., 2005). These functional improvements in PAS‐treated females occurred alongside the attenuation of fiber‐level atrophy observed histologically, suggesting a morphological basis for the improved contractile performance. However, fiber CSA is one of several determinants of muscle force output, and whether additional factors such as excitation‐contraction coupling or Ca^2+^ sensitivity contributed to the functional improvements observed in PAS‐treated females remains to be determined. These functional improvements were accompanied by greater citrate synthase activity in the casted gastrocnemius of PAS‐treated females, with no corresponding difference in males, suggesting that PAS increased mitochondrial content in females. Whether greater mitochondrial content contributes to the preservation of fiber size and force in females or simply accompanies it cannot be resolved here, but the association identifies oxidative capacity as a reasonable starting point for mechanistic investigation. Notably, PAS‐treated males did not exhibit improved absolute force or RFD, consistent with the largely female‐specific fiber‐size response in the soleus, further supporting the coherence of the sex‐specific response across morphological and functional outcomes.

In contrast to the force outcomes, PAS‐treated males demonstrated markedly higher fatigue resistance compared to VEH‐treated males, while females showed no treatment effect. This divergence suggests that PAS may influence different aspects of muscle physiology in males and females and that functional preservation with PAS is outcome dependent. Fatigue resistance is closely linked to oxidative capacity, mitochondrial function, and sarcoplasmic reticulum Ca^2+^ handling within the muscle fiber (Allen et al., 2008; Schiaffino & Reggiani, 2011). In this context, succinate, one of the two components of PAS and a widely studied mitochondrial substrate and signaling metabolite (Giorgi‐Coll et al., 2017; Xu et al., 2021; Yuan et al., 2017), is a particularly compelling candidate for mediating the improved fatigue resistance observed in males. Succinate has been shown to benefit skeletal muscle energetics by increasing oxidative phosphorylation, aerobic enzyme activity, and myosin heavy chain I expression while promoting mitochondrial biogenesis and content, adaptations that would be expected to enhance oxidative capacity and fatigue resistance (Ismaeel et al., 2023; Wang et al., 2020). However, that citrate synthase activity was unchanged in PAS‐treated males suggests any such benefit reflects mitochondrial function or substrate supply rather than an expansion of mitochondrial content. Whether oral PAS supplementation engaged these mitochondrial mechanisms preferentially in aged male muscle remains an important mechanistic question for future investigation.

The functional findings of this study are particularly noteworthy given that existing countermeasures have largely failed to preserve muscle function during disuse in older populations. Neuromuscular electrical stimulation, although widely applied, produced no improvement in maximal muscle strength, muscle fiber cross‐sectional area, or stair‐climbing power in elderly postoperative patients over 12 weeks (Suetta et al., 2008). Even when electrical stimulation is combined with protein supplementation during bed rest in older adults, functional deficits persist despite maintained muscle mass (Reidy et al., 2017). Protein and amino acid supplementation have similarly shown no significant effect on muscle strength or function during immobilization (Dirks, Wall, Nilwik, et al., 2014; Hughes et al., 2024). The sex‐specific effect of PAS supplementation on contractile function preservation points to a potentially distinct mechanism of action worthy of further investigation. The findings of the present work underscore the potential of microbial‐derived metabolites like PAS as a novel therapeutic avenue for maintaining functional capacity in aged muscle during disuse.

### 4.4 Limitations and future directions

Several limitations of the present study should be acknowledged. The study was largely confined to phenotypic and functional outcomes, and the index of mitochondrial content we included cannot resolve the pathways through which PAS acts; future studies should incorporate targeted mechanistic and molecular endpoints to elucidate the pathways underlying the sex‐specific functional responses to PAS observed in the present work. The effects of PAS on food intake and body composition were not assessed; although body mass did not differ by treatment, shifts in fat and lean mass cannot be excluded. Additionally, because only the casted soleus was assessed ex vivo, we could not determine the effect of casting on contractile function or whether PAS fully restores it. Moreover, disuse alters the gut microbiome (Alvaro‐Fuss et al., 2026; Yu et al., 2025); whether age and disuse interact to lower endogenous PAS availability, thereby contributing to muscle loss, remains to be determined. Finally, future work should examine whether PAS enhances the recovery of muscle size and function during post‐immobilization rehabilitation, as potentiating the response to remobilization would further strengthen its translational relevance as a therapeutic strategy for disuse atrophy in aging. Ultimately, studies treating sex as a primary biological variable and directly comparing varying doses of PAS in aged and young animals will be critical for establishing the full therapeutic potential and mechanistic profile of PAS in the context of disuse atrophy.

## 5 CONCLUSION

In aged mice subjected to 10 days of hindlimb immobilization, PAS produced a partial preservation of muscle size such that casted gastrocnemius mass and fiber cross‐sectional area in both the soleus and plantaris were greater in PAS‐ than VEH‐treated mice, with the largest effect in the soleus of females. PAS also augmented soleus contractile function in a sex‐specific manner, with females exhibiting greater absolute force and RFD, and males greater fatigue resistance. Greater citrate synthase activity in the casted gastrocnemius paralleled the morphological and functional benefits noted in PAS‐treated females. Together, these findings suggest that microbial‐derived exerkines attenuate disuse‐induced muscle atrophy and functional loss in a sexually dimorphic manner, highlighting the importance of considering sex as a biological variable in the development and evaluation of therapeutic strategies targeting disuse atrophy in aging.

## AUTHOR CONTRIBUTIONS

**Benjamin I. Burke:** Conceptualization; data curation; formal analysis; funding acquisition; investigation; methodology; project administration; supervision; validation; visualization. **Jainil Daredia:** Data curation; formal analysis; investigation; methodology; visualization. **Taylor R. Valentino:** Conceptualization; supervision. **Ahmed Ismaeel:** Conceptualization; supervision. **Yuan Wen:** Conceptualization; methodology; project administration; resources; software; supervision. **John J. McCarthy:** Conceptualization; funding acquisition; supervision.

## FUNDING INFORMATION

This work was supported by R01AR084282 to JJM from NIAMS and F31AG087618 to BIB from NIA.

## CONFLICT OF INTEREST STATEMENT

YW is the owner of MyoAnalytics, LLC. TRV, AI, YW, JJM, and BIB are founders of MyoBiota, Inc. and hold equity in the company. Neither company provided funding, equipment, or materials for this work, nor did either have any role in the study design, data collection, data analysis, interpretation of the results, or preparation of the manuscript. TRV, YW, JJM, and BIB are named inventors on patent application WO2025250747 which relates to the metabolites studied here.

## ETHICS STATEMENT

All animal procedures were approved by the Institutional Animal Care and Use Committee at the University of Kentucky (2018‐3005).

## Data Availability

All data sets generated during the course of this study will be made available upon reasonable request.
